# The application of pangenomics and machine learning in genomic selection in plants

**DOI:** 10.1002/tpg2.20112

**Published:** 2021-07-20

**Authors:** Philipp E. Bayer, Jakob Petereit, Monica Furaste Danilevicz, Robyn Anderson, Jacqueline Batley, David Edwards

**Affiliations:** ^1^ School of Biological Sciences and Institute of Agriculture University of Western Australia Perth WA Australia

## Abstract

Genomic selection approaches have increased the speed of plant breeding, leading to growing crop yields over the last decade. However, climate change is impacting current and future yields, resulting in the need to further accelerate breeding efforts to cope with these changing conditions. Here we present approaches to accelerate plant breeding and incorporate nonadditive effects in genomic selection by applying state‐of‐the‐art machine learning approaches. These approaches are made more powerful by the inclusion of pangenomes, which represent the entire genome content of a species. Understanding the strengths and limitations of machine learning methods, compared with more traditional genomic selection efforts, is paramount to the successful application of these methods in crop breeding. We describe examples of genomic selection and pangenome‐based approaches in crop breeding, discuss machine learning‐specific challenges, and highlight the potential for the application of machine learning in genomic selection. We believe that careful implementation of machine learning approaches will support crop improvement to help counter the adverse outcomes of climate change on crop production.

## Core Ideas

- Genomic selection (GS) has been successful in plant breeding, leading to increased yield.
- Machine learning has been applied in GS based on the ever‐growing amount of genomic data.
- Some machine learning outcomes are difficult to assess for plant breeders.
- Pangenome references are a valuable resource for GS.
- Novel methods assist in interpreting the outcomes of machine learning algorithms.

**Abbreviations**
ANN: artificial neural network
BLUP: best linear unbiased prediction
GBLUP: genomic best linear unbiased prediction
LD: linkage disequilibrium
LIME: local interpretable model‐agnostic explanation
NFL: no free lunch
QTL: quantitative trait loci
SNP: single nucleotide polymorphism
TGBLUP: Bayesian threshold genomic best linear unbiased prediction

## 1 INTRODUCTION

Plant selection is one of the fundamental structural processes in human history, supporting mankind's change in lifestyle at the beginning of the Neolithic (Bocquet‐Appel, 2011). Subsequent research and breeding has led to large increases in yield. These advances are driven by technological developments ranging from quantitative trait loci (QTL) mapping to whole‐genome sequencing. These technological improvements have enabled genomic selection, where genomic markers are linked with higher‐yielding plants enabling selection of higher‐yielding varieties in the breeding process. At the same time, industrial output has led to an increase in global temperatures in conjunction with changing weather patterns, commonly known as ‘climate change’ (Pachauri & Meyer, 2014), which has the potential to significantly alter the agricultural landscape.

Modeling yield growth shows that yield trends cannot keep up with predicted global population growth (Ray et al., 2013). This trend will be exacerbated by loss of yield as a result of climate change. Rising temperatures and extreme weather patterns in recent years demonstrate that the effects of climate change have already started (Mbow et al., 2017; NOAA National Centers for Environmental Information, 2020). Therefore, it is paramount that breeding efforts adapt to cope with these changing conditions. However, breeding new cultivars takes time; modeling efforts for all major cereals under optimum and water‐limited conditions show that overcoming the production gap of cereals will take decades rather than years (Hall & Richards, 2013). New approaches unlocking a more stable and faster breeding process across the major crops are needed to face challenges brought on by population growth and climate change.

## 2 PLANT PANGENOMES

Plant genomics is gradually moving away from single‐reference genome assemblies toward pangenomes, which reflect the genomic content of a species or family rather than an individual (Bayer et al., 2020; Danilevicz et al., 2020 ; Golicz et al., 2020; Golicz et al., 2016a; Hurgobin & Edwards, 2017). This trend started in 2016 with the publication of the first plant pangenome in *Brassica oleracea* L. (Golicz et al., 2016b), which identified an additional 2,154 gene models that were not present in the reference genome assembly and found that 20% of all genes in *B. oleracea* are variable genes that were not present in at least one individual of the cohort. Other pangenomes in plants have identified thousands of new gene models, with variable genes making up to 40% of gene content in hexaploid bread wheat (*Triticum aestivum* L.) (Montenegro et al., 2017). Currently, pangenomes have been published for bread wheat (Montenegro et al., 2017), canola (*Brassica napus* L. subsp. *napus*] (Hurgobin et al., 2018), tomato (*Solanum lycopersicum* L.) (Gao et al., 2019), sesame (*Sesamum indicum* L.) (Jingyin Yu et al., 2019), pigeonpea (*Cajanus cajan* L. Huth) (Zhao et al., 2020), soybean [*Glycine max* (L.) Merr.] (Liu et al., 2020), apple (*Malus domestica* Borkh.) (Sun et al., 2020), and barley (*Hordeum vulgare* L.) (Jayakodi et al., 2020). Development of these pangenomes has resulted in valuable insights into the history of plant domestication and breeding.

**Core Ideas**

- Genomic selection (GS) has been successful in plant breeding, leading to increased yield.
- Machine learning has been applied in GS based on the ever‐growing amount of genomic data.
- Some machine learning outcomes are difficult to assess for plant breeders.
- Pangenome references are a valuable resource for GS.
- Novel methods assist in interpreting the outcomes of machine learning algorithms.

Although they represent considerable genomic diversity, plant pangenomes have not been used widely in genomic selection. To date, only a few of the published plant pangenomes have linked the genomic data with phenotypes of interest. The tomato pangenome enabled the identification of a rare allele linked with fruit taste that had been selected against during domestication (Gao et al., 2019). In pigeonpea, presence or absence of three variable genes showed a statistically significant association with seed weight (Zhao et al., 2020). In barley, a 16.7‐kb‐large deletion containing the *NUD* gene was associated with naked barley lines, where lemma can be easily removed from grains (Jayakodi et al., 2020). Future pangenome studies will examine gene presence or absence across larger populations including wild relatives of crops (Khan et al., 2020).

Markers on large presence or absence variants are partially not captured by linkage disequilibrium (LD) with markers on the reference genome. For example, Yao et al. (2015) used LD to place novel contigs not present in the reference genome assembly. For 30.1% of newly assembled contigs, no position could be found on the reference genome based on LD with reference genome single nucleotide polymorphisms (SNPs). Phenotypes associated with these novel unplaced SNPs will not be discovered using reference‐genome‐based genomic selection approaches. In plants, genomic selection approaches therefore need to incorporate pangenomes to find all possible associations.

## 3 CURRENT STATUS OF GENOMIC SELECTION IN CROP BREEDING

One of the early advances developed to accelerate the breeding process was the calculation of breeding values using best linear unbiased prediction (BLUP) (Henderson, 1975) based on phenotypic data, which have since become a standard tool in genomic selection because of the increasing availability of genomic and phenotypic data. The development of molecular genetic markers led to the assessment of QTL (Geldermann, 1975) where regions in the genome are associated with phenotypes. Marker‐assisted selection was subsequently established, which combines breeding values and QTL into a statistical framework to mathematically estimate breeding values or to prescreen populations for specific traits of interest such as a specific disease resistance (Fernando & Grossman, 1989). Genomic selection, introduced in 2001, uses a prediction equation based on genome‐wide DNA markers to predict the effect of each marker on a trait (Meuwissen et al., 2001). One basic assumption of genomic selection is that there is sufficient LD between markers, which means that DNA markers evenly spread in the genome will capture the majority of genetic variation.

Many statistical methods have been proposed and implemented for genomic selection when assuming additive effects. The most commonly used methods are ridge‐regression BLUP, BayesA, BayesB (Meuwissen et al., 2001), BayesC (Habier et al., 2011 ), Bayesian Lasso (Park & Casella, 2008), reproducing kernel Hilbert space (Gianola et al., 2006 ), and genomic BLUP (GBLUP) (VanRaden, 2008). All of these methods are based on different assumptions about the input data. For example, the Bayesian methods allow most SNPs to have independent variance, while GBLUP assumes that all SNPs contribute equally to the phenotype. Many variants of these methods have been proposed, for example, kinship‐adjusted multiple‐loci BLUP (Yin et al., 2020) uses methods developed in machine learning to optimize parameters of a linear mixed model while adjusting for population structure.

Genomic selection has been successfully applied in crops (Heffner et al., 2009). One of the earliest studies using genomic selection in plants used genotyping‐by‐sequencing in bread wheat to estimate breeding values for grain yield (Poland et al., 2012). Other studies linked genome‐wide markers with grain yield in rice (Spindel et al., 2015), seed oil content and oil yield in canola (Jan et al., 2016 ), or with yield under drought‐conditions in chickpea (Y. Li et al., 2018).

A barrier to the successful application of genomic selection in crops is their highly complex genome (Gregory et al., 2007). Polyploid crop genomes display significant amounts of presence or absence variation as a result of homeologous recombination, where some genomic regions are duplicated between homeologous chromosomes while the region of insertion is usually lost. Homeologous recombination is a common cause of gene loss in canola (Hurgobin et al., 2018) and bread wheat (Montenegro et al., 2017), with gene loss ranging from 16 (Golicz et al., 2016) to 40% (Montenegro et al., 2017). These complex rearrangements and variable genes are not incorporated in standard genomic selection approaches, which usually focus on SNPs, and this may partially explain the moderate success of genomic selection in crops. Extending the types of markers past SNPs may increase their prediction accuracy. For example, treating missing alleles as deleted alleles, instead of removing these alleles from the data, has been shown to increase prediction accuracy as this efficiently models gene presence or absence variations (Gabur et al., 2018, 2020). The full genetic diversity present in a species can only be assessed by establishing pangenomes before applying genomic selection in order to use a broader extent of a species’ markers in the model training process.

## 4 MACHINE‐LEARNING‐BASED GENOMIC SELECTION

Standard genomic selection methods have several drawbacks. One of the biggest problems of classic genomic selection approaches is that most of these methods do not consider nonadditive effects such as epistasis or genomic imprinting (Varona et al., 2018). Furthermore, these methods usually do not calculate interactions between genotypes (Jiang & Reif, 2015), which is especially important in polyploid organisms where nonadditive effects can have a large impact on phenotypes (Endelman et al., 2018; Hunt et al., 2020; Jackson & Chen, 2010). One potential solution is to use novel machine learning and deep learning methods, which can model minor nonadditive effects as well as interactions between phenotypes or between genotypes. In recent years, advanced machine learning methods, such as random forests or artificial neural networks (ANNs), have been applied in genomic selection contexts. One study compared many standard genomic selection methods and machine learning methods in wheat, barley, *Arabidopsis thaliana* (L.) Heynh., and maize (*Zea mays* L.) datasets (Heslot et al., 2012). Performance of these methods differed between datasets with no method outperforming all other methods in all datasets. Another study compared multi‐layer perceptrons, support vector machines, and a variety of Bayesian threshold genomic BLUP (TGBLUP) to apply genomic selection in wheat datasets (Montesinos‐López et al., 2019a); TGBLUP outperformed the other two models in four of the seven datasets.

One upside of machine learning methods is that unlike classic genomic selection algorithms, they can be trained using more complex data representations, for example, multiple mixed phenotypes, to model the interaction between phenotypes. One such study used a standard densely connected ANN to analyze multiple traits with mixed phenotypes finding a modest gain in prediction accuracy when using continuous phenotypes compared with an ANN trained without mixed phenotypes (Montesinos‐López et al., 2019b).

Machine learning methods were recently applied in allopolyploid strawberry (*Fragaria* ×*ananassa* Duchesne ex Rozier) and autopolyploid blueberry (*Vaccinium corymbosum* L.) (Zingaretti et al., 2020), which compared Bayesian Lasso, Bayesian ridge regression, and a custom implemented convolutional neural network to predict five yield and fruit quality‐related traits. These methods performed similarly except for culled fruit prediction, where the convolutional neural network outperformed the other methods by 20%.

**FIGURE 1:**
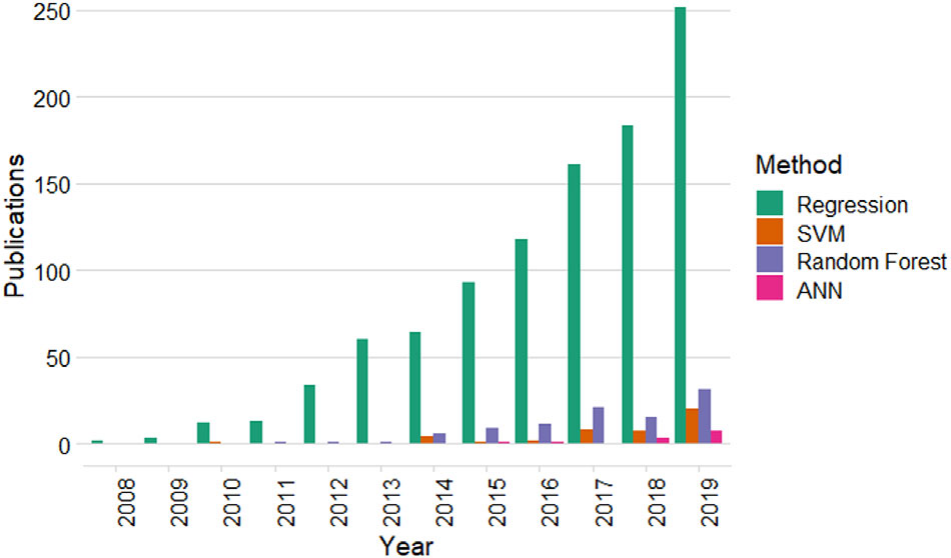
Number of publications from 2008 to 2019, inclusive, mentioning regression, support vector machines, random forest, and artificial neural networks in the context of genomic selection in crops showing that in the past five years, machine learning methods have been increasingly applied in genomic selection. Europe PMC (Levchenko et al., 2018) was queried using europepmc v0.4 (Jahn, 2020) with the following search terms: ‘(regression) AND (“genomic selection”) AND (crop*)’, ‘(“random forest”) AND (“genomic selection”) AND (crop*)’, ‘(“support vector machine”) AND (“genomic selection”) AND (crop*)’, and ‘(“artificial neural network”) AND (“genomic selection”) AND (crop*)’

Regression‐based models still form the majority of publications in the literature on genomic selection, but recent years have seen an uptake of machine‐learning‐based models (Figure 1). To our knowledge, no publication has combined these models with the data represented in a pangenome assembly. Machine learning using all the data present in a pangenome is a promising avenue for genomic selection, but breeders and researchers looking to apply these methods need to be careful. In the next section, we describe some of the most common pitfalls that occur when using machine learning and ways around these pitfalls.

## 5 CHALLENGES OF MACHINE‐LEARNING‐BASED GENOMIC SELECTION AND POTENTIAL SOLUTIONS

Machine learning libraries, such as the Python‐based scikit‐learn (Pedregosa et al., 2011), the R‐based caret (Kuhn, 2008), and deep learning libraries, such as TensorFlow (Abadi et al., 2015), Keras (Chollet, 2018), and PyTorch (Paszke et al., 2019), are well‐documented, but many invisible pitfalls remain. In this section, we present some of these pitfalls and their potential solutions.

### 5.1 Population structure modeling

Linking markers to phenotypes can suffer from spurious associations because of population structure when linking genetic markers to phenotypes, as some markers randomly appear more often by chance in some populations (Daetwyler et al., 2012). In genomic selection, several methods are being used to control population structure, most commonly a mixture of principal components analysis and a kinship matrix are used as covariates in the calculations (Jianming Yu et al., 2006). These methods work by either reducing the data into a few components (principal components analysis) or by calculating an individual‐by‐individual matrix numerically representing how closely related a pair of individuals is (kinship matrix). In most modern machine learning methods, however, measurements of kinship are not automatically calculated, as these machine learning methods are developed for a wide range of applications. Users of such methods must manually account for population structure when using modern machine learning methods, as they cannot rely on the method to independently model kinship relations. For example, users should manually calculate principal components and add them to the input data.

### 5.2 Machine learning interpretability

One of the main outcomes of classic genomic selection is a ranking of genomic variants linked with a phenotype. The ranking is then used in the breeding process to maximize breeding gains. Most machine learning methods, however, are black‐box methods, which means that researchers do not easily know which genetic markers are of importance to phenotype prediction outcomes. Machine learning models usually make thousands or millions of decisions, while deep learning models usually contain millions of model weights. It is not feasible for researchers to manually assess these decisions or model weights.

In recent years, a new machine learning research field has focused on the interpretability of machine learning. These are additional methods, which use either carefully crafted test data or additional mathematical procedures to assess, feature importance in the machine learning implementation (Molnar, 2020). In a genomic selection context, these new methods can be used to rank genetic markers based on the strength of the association with the phenotype in the model. Some machine learning methods have in‐built additional methods to quantify the impact of each feature included in the ‘learning’ process. The tree‐based XGBoost package (Chen & Guestrin, 2016) calculates three different sets of scores for each feature (column) in the input data (https://xgboost.readthedocs.io/en/latest/R‐package/discoverYourData.html). These three scores are based on how often a feature is used in the decision tree (frequency or weight), how often a feature is used in the decision tree normalized by the number of decisions (coverage), and the improvement of prediction accuracy brought by features in the branch (gain). These scores allow for a rudimentary ranking of feature importance. However, they also have drawbacks, as these are often simply a number of decisions made in the entire decision tree, the range of possible scores is different for each model, making a comparison of different XGBoost models impossible.

Some types of scores can be confusing to novice users. For example, a feature with many potential values, such as country of origin or temperature, will be used many times in the decision tree, as many potential questions can be asked about this feature. In the case of temperature, the model may learn to check whether the temperature is higher than 10 °C and lower than 12 °C, then it may learn to check whether the temperature is higher than 12 °C and lower than 13 °C and so on, leading to many possible questions. This means that in the score class ‘frequency,’ which counts how often a feature appears in the decision tree, the temperature feature will be highly ranked as many decision questions can be asked. In reality, however, the temperature may not have a large impact on the phenotype in question, but to the untrained eye, a large ‘frequency’ score can make this particular feature appear important.

To overcome the drawbacks of model‐specific interpretability more generalizable methods have been invented. One approach is to use Shapley values, which originated in game theory (Shapley, 1953; Štrumbelj & Kononenko, 2014). In Shapley values, each feature of the input data is treated as a player in a game where the outcome is the model's prediction. Each player is removed from the dataset and the average change in prediction outcome if the player (the data feature) is added to the game is calculated. This average change is called the Shapley value, which ranks feature importance by calculating all potential combinations of data, calculating how each removed or added combination changes the predictions, averaging these changes into per‐feature Shapley values, and then ordering the Shapley values numerically to infer a ranking of feature importance.

Another method to rank feature importance is called local interpretable model‐agnostic explanations (LIMEs) (Ribeiro et al., 2016). The LIME method does not remove players (data features) but instead, first chooses a specific area of values (neighborhood) and then perturbs the input data in that area to see how predictions of the model change. A simple linear model is trained on the perturbed data of a prechosen neighborhood in the input data and the prediction outcomes of the larger, more complex model. The simple linear model will learn which changes in the input data cause larger changes in the larger, more complex model and, therefore, which features of the input data have a stronger impact on model predictions. One drawback of LIME is that the input data neighborhood has to be very carefully chosen as the size of the neighborhood can have a strong impact on LIME's chosen explanations (Molnar, 2020).

To combine Shapley values and LIME, Shapley additive explanations values were introduced (Lundberg & Lee, 2017), which combine the approach of Shapley values and LIME into a new method so that the Shapley value is represented by a linear model. As these values are calculated for the entire input dataset, no choice of neighborhood is required. This allows for powerful interactive visualizations, where, for example, all Shapley values for a range of features can be plotted as dots, where each dot is colored using the range of the second feature.

To our knowledge, nobody has applied Shapley values, LIME, and Shapley additive explanations in genomic selection contexts. We believe that given the power of machine learning interpretability and the power of more complex data representation, machine learning methods will greatly outperform traditional regression‐based approaches in genomic selection.

### 5.3 The parameter optimization problem

Unlike classic genomic selection methods, the performance of machine learning methods is highly dependent on hyperparameter settings. The potential space of hyperparameters is usually too large to be traversed manually. For example, the XGBoost package has more than 15 booster hyperparameters (Chen & Guestrin, 2016), leading to potentially millions of possible combinations of hyperparameters, all of which need optimization. Some parameters have a stronger impact on the outcome of the model than others. For example, in XGBoost the number of decision trees used (n_estimators) controls how complex the resulting model is, which usually has a stronger impact on the model outcomes than other parameters.

Sometimes, insufficient optimization can explain why more complex methods perform worse when linking phenotypes to genotypes. For example, in B. Li et al. (2018), only two XGBoost parameters were optimized, which may explain why in this study, XGBoost performed worse than a gradient boosting machine.

Some parameters can be optimized using rules of thumb. For example, XGBoost's ‘scale_pos_weight’ parameter, which controls how imbalanced targets are treated, is usually calculated by dividing the number of measurements of the negative class by the number of measurements of the positive class (https://xgboost.readthedocs.io/en/latest/parameter.html). However, there is no published comprehensive evaluation of this rule, so there may be many exemptions to it.

The most commonly used method to optimize the hyperparameter space is called a grid search where the user defines a grid or a range of parameters and a training goal, for example, to maximize the area under the receiver operating characteristic curve. The machine learning software then iterates over all possible combinations of parameters to find the best‐performing combination of parameters. This is a time‐costly approach, as potentially millions of parameters have to be tested.

One alternative approach to hyperparameter optimization is Bayesian optimization such as implemented in scikit‐optimize (Head et al., 2020). Bayesian hyperparameter optimization starts at the same point as grid search with a user‐defined hyperparameter space. Unlike grid search, Bayesian optimization treats the landscape of hyperparameter as a kind of fitness space with ‘valleys’ where all parameter combinations lead to worse outcomes and ‘hills’ where all parameter combinations lead to better outcomes. By ignoring ‘valleys’ and focusing on ‘hills,’ Bayesian hyperparameter optimization is usually faster than grid search.

### 5.4 Class imbalance

When applying these hyperparameter methods, breeders and researchers need to be careful when choosing the optimization goal. In many, or most, biological datasets, the outcome variable is highly imbalanced, a common problem called class imbalance. For example, when training a predictor involving a relatively rare disease, perhaps 95% of measurements may be from unaffected individuals, while 5% are from affected individuals. A model that has learned to only predict unaffected status will therefore have an accuracy of 95%. Machine learning users, therefore, have to carefully select the target measure of the optimization steps while critically evaluating prediction outcomes. One approach is to use several different learning performance measurements such as the *F*‐score or, when predicting classes, the Matthews correlation coefficient (Matthews, 1975). Users of these methods are advised to apply and interpret these measurements carefully.

### 5.5 The ‘No Free Lunch theorem’ problem

In statistics and machine learning, one of the most famous theorems is the ‘no free lunch (NFL) theorem’ (Wolpert, 1996; Wolpert & Macready, 1997). The NFL theorem says that if two nonspecialized models are used on the same data, it is not possible to predict which one will perform better. The work of model calculation, training, or evaluation always has to be carried out—there is no free lunch.

This theorem holds only partially when comparing classic genomic selection approaches and machine learning, as the theorem specifically mentions general models not built for any special tasks such as random forests or linear regressions. Classic genomic selection approaches, however, rely on highly specialized models that use components specifically invented for genomic contexts such as, for example, kinship matrices. The NFL theorem does hold however when applying machine learning or deep learning methods in a genomic selection context.

No machine learning solution offers a shortcut for all possible problems. As a consequence, practitioners have to try several methods for different datasets, leading to automatic bias, as every practitioner has their own set of favorite methods. As another consequence, one method may just perform very well with one dataset of a training population by chance while it performs poorly with other datasets of the same training population, for example, from a different year of measurement.

Some methods to work around these problems exist but there is no one perfect solution. One solution is to always use a carefully generated holdout dataset from, for example, an additional year of phenotype measurements (Dwork et al., 2015). These holdout datasets are not used in the training or testing process, they are only used once all candidate models have been optimized and trained. Poor performance in the holdout dataset implies that the candidate model has not learned to generalize or that the initial training dataset does not contain the full range of possible phenotypes.

Meta learning methods (Lemke et al., 2015 ), such as AutoML (He et al., 2019 ) or TPOT (Olson & Moore, 2016), were formed in reaction to the NFL theorem. These methods form an additional layer on top of machine learning methods. They automatically evaluate a range of different machine learning methods and their associated hyperparameter spaces, or they automatically evaluate a range of different ANN architectures. As such, meta learning methods are not a solution to the NFL theorem but a necessary outcome. Meta learning methods are relatively new and, to our knowledge, have not been used in a genomic selection context.

## 6 CONCLUSIONS

Pangenomics and machine learning are increasingly being applied to support genomic prediction in plants. Pangenomics offers an avenue to capture the complete gene content of a species, avoiding gaps where the reference genome is significantly different from the lines under study. The increase in phenotyping and pangenome genotyping data provide large amounts of data that are suitable for machine‐learning‐based genomic selection approaches. Here we summarize the opportunities and challenges when applying machine learning methods in the context of genomic prediction providing an introduction for researchers looking to apply these methods in their research.

## CONFLICT OF INTEREST

The authors declare no conflict of interest.

## AUTHOR CONTRIBUTIONS

Philipp E. Bayer: Investigation; Writing‐original draft; Writing‐review & editing. Jakob Petereit: Investigation; Writing‐original draft; Writing‐review & editing. Monica Furaste Danilevicz: Investigation. Robyn Anderson: Investigation. Jacqueline Batley: Supervision, Writing‐review & editing. David Edwards: Project administration; Supervision; Writing‐original draft; Writing‐review & editing.
